# Detection of Old and New World Relapsing Fever Borreliae in *Ornithodoros* Ticks Collected from Warthog Burrows in Zambia

**DOI:** 10.3390/microorganisms11010200

**Published:** 2023-01-12

**Authors:** Yongjin Qiu, Herman M. Chambaro, Kozue Sato, David Squarre, Edgar Simulundu, Masahiro Kajihara, Katendi Changula, Manyando Simbotwe, Hayato Harima, Joseph Ndebe, Ladslav Moonga, Ryo Nakao, Ayato Takada, Bernard Mudenda Hang’ombe, Hirofumi Sawa, Hiroki Kawabata

**Affiliations:** 1 Division of International Research Promotion, International Institute for Zoonosis Control, Hokkaido University, North 20 West 10, Kita-ku, Sapporo 001-0020, Japan; 2 Division of Molecular Pathobiology, International Institute for Zoonosis Control, Hokkaido University, North 20 West 10, Kita-ku, Sapporo 001-0020, Japan; 3 Virology Unit, Central Veterinary Research Institute, Ministry of Fisheries and Livestock, P.O. Box 33980, Lusaka 10101, Zambia; 4 Department of Bacteriology-I, National Institute of Infectious Diseases, Toyama 1-23-1, Shinjuku, Tokyo 162-8640, Japan; 5 FQM Trident Wildlife and Forestry Programs, P.O. Box 230022, Kalumbila 10101, Zambia; 6 Royal (Dick) School of Veterinary Studies, College of Medicine and Veterinary Medicine, The University of Edinburgh, Edinburgh EH25 9RG, UK; 7 Department of Diseases Control, School of Veterinary Medicine, The University of Zambia, P.O. Box 32379, Lusaka 10101, Zambia; 8 Macha Research Trust, Macha 10101, Zambia; 9 Division of Global Epidemiology, International Institute for Zoonosis Control, Hokkaido University, North 20 West 10, Kita-ku, Sapporo 001-0020, Japan; 10 Department of Paraclinical Studies, School of Veterinary Medicine, The University of Zambia, P.O. Box 32379, Lusaka 10101, Zambia; 11 Laboratory of Parasitology, Department of Disease Control, Faculty of Veterinary Medicine, Hokkaido University, North 18 West 9, Kita-ku, Sapporo 060-0818, Japan; 12 One Health Research Center, Hokkaido University, North 20 West 10, Kita-ku, Sapporo 001-0020, Japan; 13 International Collaboration Unit, International Institute for Zoonosis Control, Hokkaido University, North 20 West 10, Kita-ku, Sapporo 001-0020, Japan; 14 Global Virus Network, 725 West Lombard St, Room S413, Baltimore, MD 21201, USA; 15 Institute for Vaccine Research and Development, Hokkaido University, North 21 West 11, Kita-Ku, Sapporo 001-0021, Japan

**Keywords:** *Borrelia*, national park, *Ornithodoros moubata*, *Ornithodoros porcinus*, relapsing fever, TaqMan MGB, Zambia

## Abstract

Relapsing fever (RF) is an arthropod-borne disease caused by *Borrelia* spirochete, which is one of the major public health concerns in endemic regions including Africa. However, information on *Borrelia spirochetes* is limited in Zambia. Here, we investigate the *Borrelia spirochetes* harbored by *Ornithodoros* ticks in Zambian National Parks. We analyzed 182 DNA samples pooled from 886 *Ornithodoros* ticks. Of these, 43 tested positive, and their sequence revealed that the ticks harbored both Old and New World RF borreliae. This research presents the first evidence of Old-World RF borreliae in Zambia. The New World RF borreliae detected herein differed from the *Candidatus* Borrelia fainii previously reported in Zambia and were closely related to the pathogenic *Borrelia* sp. VS4 identified in Tanzania. Additionally, *Borrelia theileri* was recently reported in Zambia. Hence, at least four different *Borrelia* species occur in Zambia, and the organisms causing relapsing fever there might be more complex than previously thought. We empirically confirmed that real-time PCR with TaqMan minor groove binder probes accurately and simultaneously detected both Old and New World RF. In this manner, they could facilitate quantitative analyses of both types of RF borreliae. Subsequent investigations should endeavor to isolate the aforementioned *Borrelia* spp. and perform serosurveys on patients with RF.

## 1. Introduction

The vector-borne spirochetes of the family *Borreliaceae* currently comprise 42 named species [1]. Their natural transmission cycle is maintained between vertebrate reservoir hosts such as rodents and humans and vector arthropods such as ticks and lice. The Borrelia phylogenetic groups include Lyme disease (LD) borreliae, relapsing fever (RF) borreliae, monotreme-associated borreliae, and reptile-associated borreliae [2,3,4]. LD borreliae, monotreme-associated borreliae, and reptile-associated borreliae are transmitted by Ixodid (hard-bodied) ticks. RF borreliae are transmitted mainly by lice and Argasid (soft-bodied) ticks [5]. RF borreliae are further categorized into Old World (Afrotropic-Palearctic Ecozones) RF borreliae, New World (Nearctic Ecozone) RF borreliae, and hard tick-borne RF borreliae. They are distinguished by their endemic regions and/or the genetic lineages of the causative agents [5,6].

Northern, central, and eastern African countries are endemic regions of Old World RF borreliae, such as *Borrelia hispanica*, *B. duttonii*, and *B. crocidurae*. Studies on *Borrelia* spirochetes have been conducted in Ethiopia, Mali, Morocco, Senegal, and Tanzania [7,8,9,10]. However, only two investigations of *Borrelia* spirochetes have been reported from Zambia, a country in southern Africa. We previously reported that *Candidatus* Borrelia fainii closely related to New World RF borreliae was isolated from a patient bitten by a tick in a cave in Zambia [11]. *Candidatus* B. fainii was also detected in Egyptian fruit bats (*Rousettus aegyptiacus*) and soft ticks (*Reticulinasus (Ornithodoros) faini*) collected from the cave [11]. We also reported that *Borrelia theileri*, the causative agent of bovine borreliosis transmitted by hard ticks, was detected in wild impalas and domestic cattle in the Kafue ecosystem in Zambia [12]. On the other hand, there has been no evidence of the presence of Old-World RF borreliae in Zambia to date, whereas *Borrelia duttonii*, which is a member of the Old-World RF borreliae, is endemic in Tanzania and the Democratic Republic of the Congo neighboring Zambia [10,13].

Relapsing fever is a major public health concern in several African countries. Countermeasures against RF are urgently needed. Many cases of infection involve both adults and children, and vertical transmission of the spirochetes during the perinatal period has been reported [14]. Moreover, RF is often misdiagnosed as malaria based on its clinical symptoms. Consequently, the appropriate therapy may not be administered in these cases [15]. Therefore, it is necessary to develop and distribute appropriate diagnostic systems for the detection of RF in Africa.

Relapsing fever pathogens have broad genetic diversity. Hence, laboratory diagnoses of RF in African countries require a testing system that can detect both Old World and New World RF borreliae. Countries in the Northern Hemisphere such as the United States represent the non-endemic area of Old-World RF borreliae. Researchers there have developed a modified TaqMan minor groove binder (MGB) probe-based real-time PCR system that detects New World and hard tick-borne RF borreliae [16]. Nevertheless, it is necessary to confirm that this methodology can also detect Old World RF borreliae before it is introduced to African countries.

In the present study, we detected and genetically characterized both Old and New World RF borreliae in the soft ticks that infested warthog burrows in Zambian national parks. We also verified a modified MGB probe-based real-time PCR system initially developed by Barbour et al. (2009) [16] for the simultaneous detection of Old and New World RF borreliae.

## 2. Materials and Methods

Tick collections in the national parks were approved by the Department of National Parks and Wildlife, Ministry of Tourism and Arts, Zambia (Permission No. DNPW/8/27/1) as previously described [17,18].

**Figure 1:**
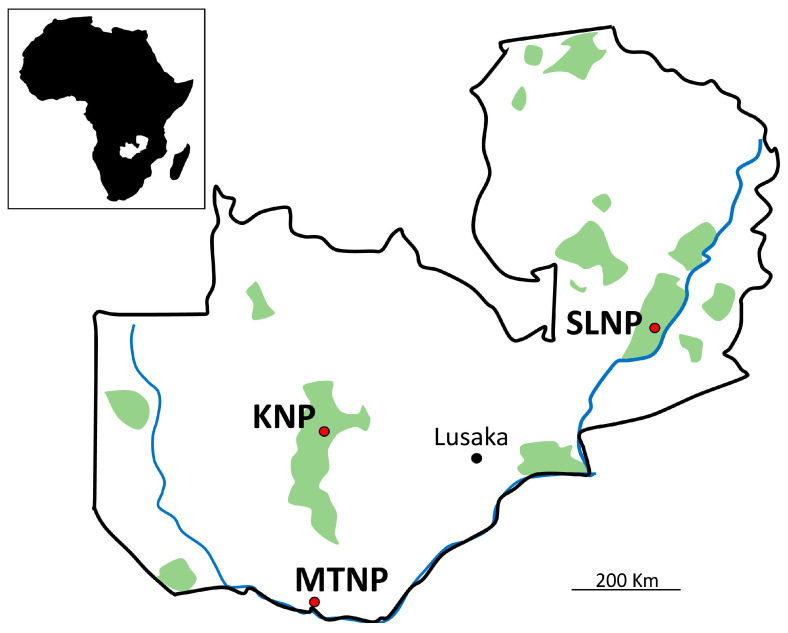
**Map of sample collection sites.** Capital city and sampling sites are displayed in black and red points. MTNP: Mosi-oa-Tunya National Park, KNP: Kafue National Park, SLNP: South Luangwa National Park.

A total of 724 *Ornithodoros moubata* ticks were recovered from African warthog (*Phacochoerus africanus*) burrows and culverts in Mosi-oa-Tunya National Park (MTNP) in April 2019 (Figure 1). Another 75 and 87 *O. porcinus* ticks were collected from African warthog burrows and culverts in Kafue National Park (KNP) and South Luangwa National Park (SLNP), respectively, in December 2019 (Figure 1). Tick species were confirmed by morphological and molecular identification in the previous studies [17,18] before being pooled for DNA extraction. Each tick pool consisted of either two or three adult ticks or six to eight nymph ticks from the same infested burrow and culvert, but the feeding status and sex of the ticks were not considered. The DNA was extracted with a 100 μL elution buffer using DNeasy® Blood and Tissue Kit (Qiagen, Hilden, Germany) according to the manufacturer’s protocol. In total, 124 *O. moubata* and 24 and 34 *O. porcinus* DNA samples were obtained from MTNP, KNP, and SLNP, respectively. These samples have been used in previous studies on African swine fever and *Rickettsia* spp. [12,17,18]. 

All samples were screened for *Borrelia* spp. via nested-PCR of the flagellin gene (*flaB*) [3]. The first PCR was performed using the BflaPAD and BflaPDU primers and the nested-PCR using the BflaPCR and BflaPBU primers, which yielded 345 bp. The composition of the reaction mixture was the same for the first- and nested-PCRs, except that 1 μL of the first PCR product was added in place of 2 μL of template DNA in the nested-PCR. The 20 μL reaction mixture contained 0.1 μL *Ex Taq* HS (Takara Bio Inc., Kusatsu, Shiga, Japan), 2 μL of 10 × *Ex Taq* buffer, 1.6 μL of 2.5 mM deoxynucleotide triphosphate (dNTP) mixture, and 200 nM of each primer. The PCR conditions were as follows: 98°C for 1 min, followed by 35 cycles of 94°C for 30 s, 50°C for 30 s, 72°C for 60 s, and a final extension at 72°C for 5 min. The positive samples were used in the subsequent PCR with the BF1 and BR1 primers targeting *Borrelia* spp. 16S ribosomal DNA (rDNA) and amplifying approximately 1.3 kbp [19]. For further characterization, positive samples from the 16S rDNA PCR were used in the subsequent PCRs targeting the glycerophosphodiester-phosphodiesterase gene (*glpQ*) and the hypoxanthine-guanine phosphoribosyltransferase gene (*hpt*). Two PCRs were conducted to obtain longer *glpQ* sequences and the first and second PCR sequences were assembled. As previously described, the glpQf1F + glpQf1R and the glpQf2F + glpQf2R primer sets were used to amplify fragments 1 (559 bp) and 2 (453 bp), respectively [20]. For *hpt*, the hptdegF and hptdegR primers were used to amplify 433 bp fragments [21]. The PCR conditions for 16S rDNA, *glpQ*, and *hpt* PCRs were the same as described above except for the annealing temperature (55°C). The sizes of all PCR products were checked by 1.5% agarose gel electrophoresis in Gel-Red™ (Biotium, Hayward, CA, USA) and visualized with a UV-transilluminator. The primers used in the PCRs are listed in Table S1. 

NucleoSpin Gel and PCR clean-up kit (Takara Bio Inc., Kusatsu, Siga, Japan) or ExoSAP-IT™ Express PCR clean-up reagent (Thermo Fisher Scientific, Auburn, AL, USA) were used to purify the PCR products. BigDye Terminator Chemistry v. 3.1 (Applied Biosystems Inc (ABI), Foster City, CA, USA) was used for cycle sequencing of all amplicons in both forward and reverse directions. The sequencing products were run on a 3130xl Genetic Analyzer (ABI) or a 3500 Genetic Analyzer (ABI) according to the manufacturer’s instructions. The 5′ and 3′ ends of the sequences were trimmed with GENETYX v. 9.1 (GENETYX Corporation, Tokyo, Japan). The sequences obtained herein were compared with those in public databases with BLASTn. Phylogenetic analysis was conducted with MEGA X [22], and ClustalW was used to align the sequences with those of closely related organisms deposited in the database (DDBJ/EMBL/GenBank). The maximum likelihood (ML) method and the Kimura-2 parameter model were used to generate phylogenetic trees based on each gene. The representative *Borrelia* spp. DNA sequences obtained in the present study are available in the GenBank database and their accession numbers are listed in Table S2 (Accession nos. LC741320- LC741337).

To verify MGB probe-based real-time PCR for simultaneous detection of different RF borreliae, multiplex real-time PCR was performed on the positive samples according to the method of Barbour et al. (2009) [16] with the modification of probes [23]. The real-time PCR conditions were as follows: 95 °C for 10 s followed by 45 cycles at 95 °C for 5 s and 60 °C for 35 s. Amplification was performed in an Applied Biosystems 7500 Real-Time PCR system (Thermo Fisher Scientific, Waltham, MA, USA). The 25 µL reaction mixture was prepared by adding 1 µM of each PCR primer (16SRT-F and 16SRT-R; Table S3) and 0.24 µM of each MGB probe (NWB_VIC_1794 and OWB_FAM_1795; Table S3) to Luna Universal probe qPCR Master Mix (New England Biolabs (NEB), Ipswich, MA, USA) according to the manufacturer’s instructions. MGB probes were purchased from Thermo Fisher Scientific. For the analysis of the PCR results, the threshold line was set to 0.2 to avoid detecting nonspecific fluorescence.

The plasmids pBDrrs and pBFrrs were used as control DNA sources to evaluate the copy number via 16S rRNA gene-based real-time PCR. Regions of the 16S rRNA gene of *B. duttonii* strain Ly and *Ca*. B. fainii strain Qtaro were amplified by PCR using 16SRT-F and 16SRT-R primer set (Table S3). The 70 bp DNA amplicons were independently cloned into the pCR4-TOPO plasmid vector (Thermo Fisher Scientific). The plasmid DNA was amplified in *Escherichia coli* strain DH5α (Nippon Gene, Tokyo, Japan), as previously described [24]. The aforementioned plasmids were purified using the PureYield Plasmid Miniprep System (Promega, Madison, WI, USA). The plasmid concentration was measured with NanoDrop Lite (Thermo Fisher Scientific). The plasmids containing partial 16S rDNA sequence of *B. duttonii* strain Ly and *Ca*. B. fainii strain Qtaro were labeled as pBDrrs and pBFrrs, respectively. Each plasmid was serially diluted and used as a control DNA source for the qPCR assay.

## 3. Results

### 3.1. Screening of Borrelia with flaB Nested-PCR

In total, 39 of the 124 *O. moubata* pools from MTNP and 4 of the 34 *O. porcinus* pools from SLNP showed positive by *flaB* nested-PCR. None of the samples from KNP was positive for *flaB* nested-PCR (Table 1). The total pool prevalence in *Ornithodoros* ticks was 23.6% (43/182). The pool prevalences were 31.6% (39/124) and 11.8% (4/34) in the *O. moubata* at MTNP and the *O. porcinus* at SLNP, respectively (Table 1). 

The sequences from *O. moubata* pools had six variations labeled flaB_Types 1–6, and those from the *O. porcinus* pools had two variations labeled flaB_Types 1 and 7 (Table 2). The flaB_Type 1 sequences were obtained from the 30 *O. moubata* pools and the 3 *O. porcinus* pools. The flaB_Type 2, 4, 5, and 6 sequences were obtained from *O. moubata* pools (Sample IDs: MTNP-7, MTNP-32, MTNP-45, and MTNP-95). The flaB_Type 7 sequence was obtained from one *O. porcinus* pool (Sample ID: SLNP-19) (Table 2).

**Figure 2:**
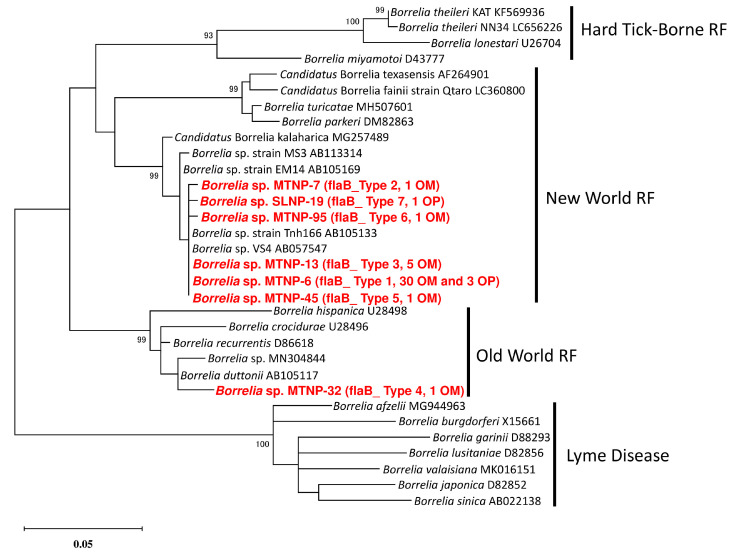
**Phylogenetic tree of the *Borrelia* spp. based on the partial sequence of the flagellin gene *flaB*.** Analyses were performed by the maximum likelihood (ML) method with the Kimura-2 parameter. The sequences generated herein are displayed in bold and red font. The flagellin sequence variations and the numbers of the samples with the same sequence are enclosed in brackets. OM and OP refer to *Ornithodoros moubata* and *O. porcinus*, respectively. Bootstrap values > 70% are based on 1000 replications and are shown beside the branches.

In the phylogenetic tree based on the partial *flaB* sequence, the flaB_Type 1, 2, 3, 5, 6, and 7 sequences were clustered together with *Borrelia* sp. VS4 (accession No. AB057547) and located in the clade of the New World RF borreliae (Figure 2). The flaB_Type 4 sequence was clustered together with *B. duttonii*, which is a member of the Old-World RF borreliae (Figure 2).

### 3.2. Analysis of 16S Ribosomal DNA

The 16S rDNA sequences were obtained from 25 and 4 positive samples of *O. moubata* in MTNP and *O. porcinus* in SLNP, respectively (Table 3). There were seven sequence variants (16S_Type 1 to 7). The 16S_Type 1 sequence was obtained from 18 and 3 of *O. moubata* and *O. porcinus* pools, respectively. The 16S_Type 2–4 and 6 sequences were from *O. moubata* pools. The 16S_Type 7 sequence from *O. porcinus* pool. The 16S_Type 5 sequence from one *O. moubata* pool (Sample ID: MTNP-32) (Table 3). 

**Figure 3:**
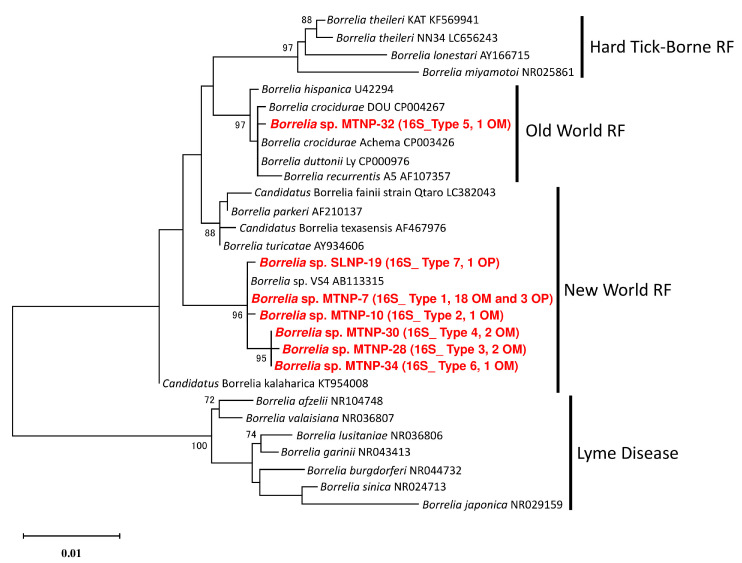
**Phylogenetic tree of the *Borrelia* spp. based on 1355 bp of 16S ribosomal DNA sequences.** The analyses were performed by the ML method with the Kimura-2 parameter. The sequences generated herein are displayed in bold and red font. The 16S rDNA sequence types and the numbers of samples with the same sequence are enclosed in brackets. OM and OP refer to *Ornithodoros moubata* and *O. porcinus*, respectively. Bootstrap values > 70% are based on 1000 replications and are shown beside the branches.

In the phylogenetic tree based on the nearly full length of the 16S rDNA sequences, the sequences from all ticks in MTNP and SLNP except single MTNP-32 were clustered together with *Borrelia* sp. VS4 and located in the clade of the New-World RF borreliae (Figure 3). The sequence from MTNP-32 was located in the clade of the Old-World RF borreliae (Figure 3).

### 3.3. Analysis of glpQ and hpt Genes

**Figure 4:**
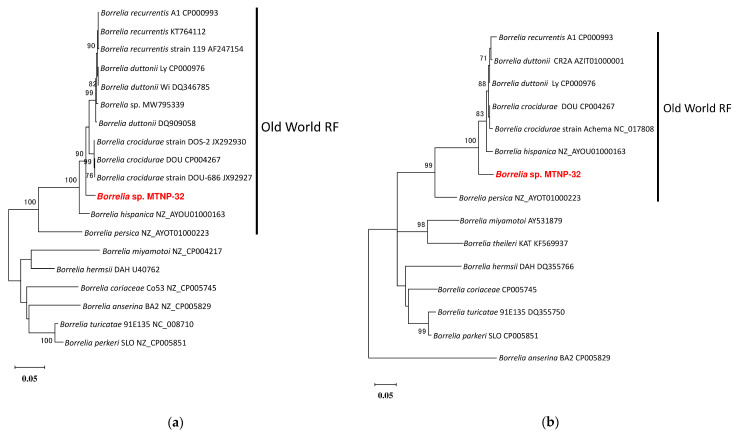
**Phylogenetic trees of *Borrelia* sp. from MTNP-32.** The analyses were performed by the ML method with the Kimura-2 parameter. Bootstrap values > 70% are based on 1000 replications and are shown beside the branches. (**a**) The tree based on 840 bp of the *glpQ* sequence; (**b**) the tree based on 388 bp of the *hpt* sequence. The sample in this study is in bold and red.

The *glpQ* and *hpt* sequences were obtained exclusively from MTNP-32. For *glpQ*, the sequence showed 97.4% (818/840 bp) identity with *B. crocidurae* (accession No. JX292930). In the phylogenetic tree based on the partial *glpQ* sequence, the sequence from MTNP-32 clustered together with the Old-World RF borreliae (Figure 4a). However, the *hpt* sequence showed 95.1% (369/388 bp) identity with *B. crocidurae* DOU (accession No. CP004267) and *B. duttonii* Ly (accession No. CP000976). In the phylogenetic tree based on the partial *hpt* sequences, the sequence from MTNP-32 clustered together with the Old-World RF borreliae (Figure 4b).

### 3.4. Real-Time PCR

**Figure 5:**
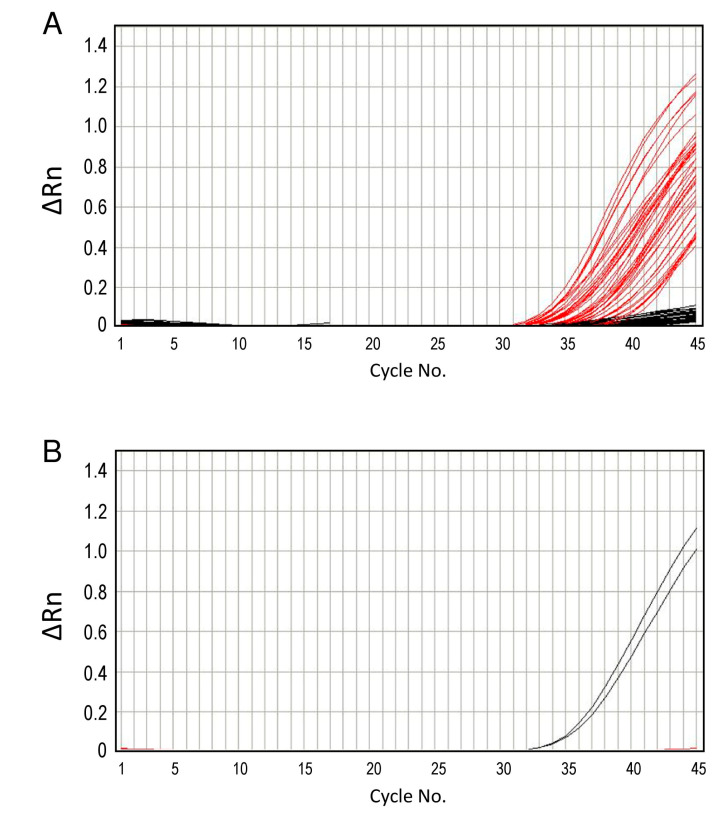
**Detection of *Borrelia* DNA from tick specimens by real-time PCR.** PCR positive by NWB_VIC_1794 (red line) and OWB_FAM_1795 (black line) probes were shown in (**A**,**B**), respectively. Each tick specimen was examined in duplicate in this study.

The 42 samples identified as positive for the *flaB* nested-PCR were used in the real-time PCR. Of these, 23 were also positive for the 16S rDNA PCR. Out of the 42 samples, 38 (90.5%) were positive for the real-time PCR (Figure 5). Four negative samples on real-time PCR were also negative for the 16S rDNA PCR. The *Borrelia* sp. from MTNP-32 was closely related to *B. duttonii* and was successfully detected only by the OWB_FAM_1795 probe designed for Old World RF borreliae. In contrast, the other 37 samples containing New World RF borrelia were detected only by the NWB_VIC_1794 probe designed for New World RF borreliae and *B. miyamotoi*. The *Borrelia* copy number was in the range of 4.3–5835 (Table S2). When *flaB* nested-PCR was set as the gold standard, the real-time PCR method used herein had 90.5% (38/42 samples) sensitivity and 100% (38/38 samples) specificity.

## 4. Discussion

In the present study, we investigated *Borrelia* spirochetes in soft ticks collected from African warthog burrows and culverts in three National Parks in Zambia. Two different *Borrelia* spp. harbored by *Ornithodoros* ticks were characterized by multiple genes and were found to belong to Old World and New World RF borreliae. We tested the sensitivity and specificity of a multiplex real-time PCR in detecting both Old World and New World RF borreliae with the newly designed probes. To the best of our knowledge, the present study is the first to report Old World RF borreliae in Zambia. 

*Borrelia duttonii* causes RF and is transmitted by *Ornithodoros moubata* complex ticks, such as *O. moubata* and *O. porcinus* in East African countries. In endemic areas in Tanzania, the rate of house infestation with *O. moubata* complex ticks is very high (up to 88%) [25]. RF caused by *B. duttonii* is one of the diseases with the highest fatality among children and pregnant women in endemic areas [26]. In the present study, a *Borrelia* sp. closely related to *B. duttonii* was detected in an *O. moubata* pool (MTNP-32) in MTNP. This *Borrelia* sp. showed high identity with *B. duttonii* on the 16S rDNA sequence. Its *flaB*, *glpQ*, and *hpt* sequences only showed 92.6 to 97.4% identity with those invalidated *Borrelia* species. Hence, it is difficult to conclude whether *Borrelia* sp., closely related to *B. duttonii,* is likely to be pathogenic to humans. Therefore, *Borrelia* sp. needs to be characterized in detail, and it is of interest to evaluate its pathogenicity in further research. 

Here, New World RF borreliae were detected from *O. moubata* and *O. porcinus* ticks collected in MTNP and SLNP, respectively. Their *flaB* and 16S rDNA sequences were similar or identical to those of *Borrelia* sp. VS4. The latter was first detected in *O. porcinus* isolated in Mvumi, central Tanzania [27]. It was then found in the blood of a febrile child living in the aforementioned area [28]. Hence, these *Borrelia* spp. belonging to the New World RF borreliae might be pathogenic to humans. Only one case of human borreliosis has been reported in Zambia [11]; however, it was caused by *Ca*. B. fainii. Therefore, the agents of human borreliosis in Zambia might be more divergent. To reveal the actual situation of human borreliosis in Zambia, further investigations such as serological surveys and screening of *Borrelia* in patients with a fever of unknown origin are warranted.

In a study conducted in central Tanzania, the total prevalence of *Borrelia* spp. in *O. porcinus* was 50% (60/120), while the prevalences of *B. duttonii* and New World RF borrelia in the *Ornithodoros* ticks were 42.5% (51/120) and 7.5% (9/120), respectively [29]. In the present study, the total pool prevalence of *Borrelia* spp. in *Ornithodoros* ticks was 23.6% (43/182). Further, *Borrelia* sp. closely related to *B. duttonii* and New World RF borreliae in *Ornithodoros* ticks had a pool prevalence of 0.5% (1/182) and 23.1% (42/182), respectively. Since ticks were pooled in the present study, directly comparing the prevalence with that in central Tanzania would not be possible. Furthermore, in the previous study in central Tanzania, the tick specimens were collected in homes, and humans were considered the major hosts. By contrast, our tick specimens were collected from warthog burrows, and warthogs were considered the hosts. Therefore, to evaluate the risk of human borreliosis in Zambian national parks, further research is required to account for the usage of the parks and the prevalence of borrelia in ticks.

Mitani et al. (2004) attempted to isolate the New World RF borreliae designated *Borrelia* sp. VS4 from *O. porcinus* in Tanzania by using a BSK-II culture medium. However, the isolation was unsuccessful [29]. There have been several reports of New World RF borreliae in Africa and Eurasia. For instance, *Ca*. B. kalaharica was detected in a febrile patient returning from southern Africa in 2016 and in *Ornithodoros savignyi* ticks in Nigeria in 2018 [30,31,32]. The *Ca.* Borrelia fainii was detected in a febrile patient and cave habituating bats and soft ticks in Zambia, and it was also detected in bats in China [11,33,34]. Only our previous study successfully isolated *Borrelia* sp. belonging to New-World RF borreliae in the Old World [11]. Thus, the isolation of other New World RF borreliae distributed in the Old World, such as *Borrelia* sp. VS4 is strongly needed to better characterize this unique borrelia group.

Here, the *glpQ* sequence was obtained only from the *Borrelia* sp. from the MTNP-32 sample. This *Borrelia* sp. belonged to the Old-World RF borreliae. The same sample was positive by PCR targeting *hpt.* By contrast, all samples containing the New World RF borreliae were negative for both *glpQ* and *hpt* PCRs. Therefore, the current primer sets targeting *glpQ* and *hpt* might be unsuitable for use on the New World RF borreliae detected herein. Real-time PCR with modified probes successfully detected both Old and New World RF borreliae with high specificity. Ten copies per reaction were sufficient for the detection. Therefore, this method might be useful for quantitative analysis of *Borrelia* spirochete in regions where both Old and New World RF are endemic.

In the present study, we detected *Borrelia* sp. belonging to the New World RF borreliae, a species different from the ones previously reported in Zambia, from *Ornithodoros* ticks. We also detected *Borrelia* sp. closely related to *B. duttonii* from the ticks. To date, only two *Borrelia* species, namely, *Ca*. B. fainii and *B. theileri*, have been reported in Zambia [11,35].

To the best of our knowledge, at least four *Borrelia* species are currently present in Zambia. The causative agents of RF in Zambia might be more complex than previously thought. Further investigations, such as isolation of the detected *Borrelia* spp., nationwide surveillance of *Borrelia* spirochetes, and serological survey in humans, are required for a better understanding of the *Borrelia* spp. and public health in the country.

## Figures and Tables

**Table 1:** **Results of borrelia screening using *flaB* nested-PCR**.

Tick Species	Sampling Location	No. Tick Collected	No. Positive (Tested Pools)	Pool Prevalence
*Ornithodoros moubata*	Mosi-oa-Tunya National Park	724	39 (124)	31.6%
*Ornithodoros porcinus*	Kafue National Park	75	0 (24)	NA
*Ornithodoros porcinus*	South Luangwa National Park	87	4 (34)	11.8%
Total		886	43 (182)	23.6%

NA: Not applicable.

**Table 2:** **Results of BLASTn search of *flaB* sequences**.

Variants	Sample IDs	Identity	Species
flaB_Type 1	MTNP-6, MTNP-8, MTNP-9, MTNP-10, MTNP-14, MTNP-20, MTNP-22, MTNP-24, MTNP-28, MTNP-29, MTNP-30, MTNP-31, MTNP-33, MTNP-34, MTNP-36, MTNP-37, MTNP-40, MTNP-41, MTNP-42, MTNP-43, MTNP-44, MTNP-49, MTNP-80, MTNP-87, MTNP-88, MTNP-93, MTNP-117, MTNP-118, MTNP-120, MTNP-122, SLNP-13, SLNP-27, SLNP-34	100% (294/294 bp)	*Borrelia* sp. VS4 (AB057547) *
flaB_Type 2	MTNP-7	99.7% (293/294 bp)	*Borrelia* sp. VS4 (AB057547) *
flaB_Type 3	MTNP-13, MTNP-89, MTNP-96, MTNP-100, MTNP-103	99.7% (293/294 bp)	*Borrelia* sp. VS4 (AB057547) *
flaB_Type 4	MTNP-32	92.6% (275/297 bp)	*B. duttonii* (AB105117)
flaB_Type 5	MTNP-45	93.9% (276/294 bp)	*Borrelia* sp. VS4 (AB057547) *
flaB_Type 6	MTNP-95	99.3% (292/294 bp)	*Borrelia* sp. VS4 (AB057547) *
flaB_Type 7	SLNP-19	99.7% (293/294 bp)	*Borrelia* sp. VS4 (AB057547) *

* *Borrelia* sp. VS4 was registered as *B. duttonii* strain TnB. However, the reference papers labeled it as *Borrelia* sp. op Type C or *Borrelia* sp. VS4, and it was detected in *O. porcinus* in the Mvumi region of Tanzania.

**Table 3:** Results of BLASTn search of 16S ribosomal DNA sequences.

Variants	Sample IDs	Identity	Species
16S_Type 1	MTNP-7, MTNP-8, MTNP-9, MTNP-13, MTNP-14, MTNP-22, MTNP-24, MTNP-29, MTNP-31, MTNP-33, MTNP-41, MTNP-42, MTNP-43, MTNP-45, MTNP-80, MTNP-88, MTNP-100, MTNP-122, SLNP-13, SLNP-27, SLNP-34	100% (1348/1348 bp)	*Borrelia* sp. VS4 (AB113315) *
16S_Type 2	MTNP-10	99.9% (1347/1348 bp)	*Borrelia* sp. VS4 (AB113315) *
16S_Type 3	MTNP-28, MTNP-37	99.7% (1344/1348 bp)	*Borrelia* sp. VS4 (AB113315) *
16S_Type 4	MTNP-30, MTNP-36	99.9% (1347/1348 bp)	*Borrelia* sp. VS4 (AB113315) *
16S_Type 5	MTNP-32	99.8% (1352/1355 bp)	*B. duttonii* strain Ly (CP000976) and *B. crocidurae* strain Achema (CP003426).
16S_Type 6	MTNP-34	99.8% (1345/1348 bp)	*Borrelia* sp. VS4 (AB113315) *
16S_Type 7	SLNP-19	99.9% (1347/1348 bp)	*Borrelia* sp. VS4 (AB113315) *

*: *Borrelia* sp. VS4 was registered as *B. duttonii* strain TnB. However, the reference papers labeled it as *Borrelia* sp. op Type C or *Borrelia* sp. VS4, and it was detected in *O. porcinus* in the Mvumi region of Tanzania.

## Data Availability

All relevant data are provided in the manuscript.

## Supplementary Materials

The supporting information can be downloaded at: https://www.mdpi.com/article/10.3390/microorganisms11010200/s1, Table S1: The primers used for PCRs in the present study, Table S2: List of positive samples with the accession number and the real-time PCR results, Table S3: Oligonucleotides used for real-time PCR in the present study.
